# Contraction influences *Per2* gene expression in skeletal muscle through a calcium‐dependent pathway

**DOI:** 10.1113/JP280428

**Published:** 2020-10-04

**Authors:** Lewin Small, Ali Altıntaş, Rhianna C Laker, Amy Ehrlich, Pattarawan Pattamaprapanont, Julia Villarroel, Nicolas J Pillon, Juleen R Zierath, Romain Barrès

**Affiliations:** ^1^ Novo Nordisk Foundation Center for Basic Metabolic Research, Faculty of Health and Medical Sciences University of Copenhagen Copenhagen Denmark; ^2^ Department of Physiology and Pharmacology Karolinska Institutet Stockholm Sweden; ^3^ Department of Molecular Medicine and Surgery Karolinska Institutet Stockholm Sweden

**Keywords:** calcium, clock genes, contraction, period genes, skeletal muscle

## Abstract

**Key points:**

- Exercising at different times of day elicits different effects on exercise performance and metabolic health. However, the specific signals driving the observed time‐of‐day specific effects of exercise have not been fully identified.
- Exercise influences the skeletal muscle circadian clock, although the relative contribution of muscle contraction and extracellular signals is unknown.
- Here, we show that contraction acutely increases the expression of the core circadian clock gene Period Circadian Regulator 2 (Per2) and phase‐shifts *Per2* rhythmicity in muscle cells. This contraction effect on core clock genes is mediated through a calcium‐dependant mechanism;
- The results obtained in the present study suggest that a proportion of the ability of exercise to entrain the skeletal muscle clock is driven directly by muscle contraction. Contraction interventions may be used to mimic some time‐of‐day specific effects of exercise on metabolism and muscle performance.

**Abstract:**

Exercise entrains the central and peripheral circadian clocks, although the mechanism by which exercise modulates expression of skeletal muscle clock genes is unclear. The present study aimed to determine whether skeletal muscle contraction alone could directly influence circadian rhythmicity and uncover the underlying mechanism by which contraction modulates clock gene expression. We investigated the expression of core clock genes in human skeletal muscle after acute exercise, as well as following *in vitro* contraction in mouse soleus muscle and cultured C2C12 skeletal muscle myotubes. Additionally, we interrogated the molecular pathways by which skeletal muscle contraction could influence clock gene expression. Contraction acutely increased the expression of the core circadian clock gene Period Circadian Regulator 2 (Per2) and phase‐shifted *Per2* rhythmicity in C2C12 myotubes *in vitro*. Further investigation revealed that pharmacologically increasing cytosolic calcium concentrations by ionomycin treatment mimicked the effect of contraction on *Per2* expression. Similarly, treatment with a calcium channel blocker, nifedipine, blocked the effect of electric pulse stimulation‐induced contraction on *Per2* expression. Increased calcium influx from contraction lead to binding of the phosphorylated form of cAMP response element‐binding protein (CREB) to the *Per2* promoter, suggesting a role of CREB in contraction‐induced *Per2* transcription. Thus, by dissociating the effect of muscle contraction alone from the whole effect of exercise, our investigations indicate that a proportion of the ability of exercise to entrain the skeletal muscle clock is driven directly by contraction.

## Key points

- Exercising at different times of day elicits different effects on exercise performance and metabolic health. However, the specific signals driving the observed time‐of‐day specific effects of exercise have not been fully identified.
- Exercise influences the skeletal muscle circadian clock, although the relative contribution of muscle contraction and extracellular signals is unknown.
- Here, we show that contraction acutely increases the expression of the core circadian clock gene Period Circadian Regulator 2 (Per2) and phase‐shifts *Per2* rhythmicity in muscle cells. This contraction effect on core clock genes is mediated through a calcium‐dependant mechanism;
- The results obtained in the present study suggest that a proportion of the ability of exercise to entrain the skeletal muscle clock is driven directly by muscle contraction. Contraction interventions may be used to mimic some time‐of‐day specific effects of exercise on metabolism and muscle performance.

## Introduction

The central regulation of circadian rhythm is controlled by the suprachiasmatic nucleus (SCN), a discreet component of the hypothalamus that is entrained by light and signals for many circadian behaviours, such as eating and sleeping (Hastings *et al*. 2003). In addition to receiving input from the central clock, peripheral tissues have their own molecular clocks comprising positive and negative regulatory proteins that control transcription of each other. The positive element proteins BMAL1 (*Arntl*) and CLOCK dimerize and bind to an E‐box motif in the promoter regions of NR1D1 to drive its transcription, as well as expression of the Period Circadian Regulator (*Per1*, *2* and *3*) and Cryptochrome (*Cry1* and *2*) genes. NR1D1 in turn feeds back to bind the DNA response element in the BMAL1 promoter, blocking its subsequent transcription, whereas the CRY and PER proteins translocate to the nucleus to block BMAL1 activity. This entire process forms the molecular clock in which core clock genes and proteins display oscillations in mRNA and protein abundance with a period of approximately 24 h (Gekakis *et al*. 1998; Kume *et al*. 1999; Shearman *et al*. 2000). BMAL1 and CLOCK also bind to hundreds of other E‐box motifs to regulate transcription of genes that are not classically circadian in function, including many genes involved in metabolism, such as the peroxisome proliferator‐activated receptors, sirtuins and peroxisome proliferator‐activated receptor gamma coactivator‐1α (Gerhart‐Hines & Lazar, 2015).

Similar to other peripheral tissues, the skeletal muscle has its own intrinsic clock machinery (Harfmann *et al*. 2014), where specific deletions in this machinery lead to aberrant transcription of metabolic genes. As a result, impaired physiological responses such as altered glucose handling occur (Dyar *et al*. 2014; Harfmann *et al*. 2016). Interestingly, as demonstrated in fibroblasts (Balsalobre *et al*. 1998; Yagita *et al*. 2001) and primary muscle cells (Perrin *et al*. 2018), even when taken out of an organism and disconnected from photic cues, cells cultured *in vitro* display rhythmic expression in many of the core clock genes. Important to the present study, the mouse C2C12 immortalized myoblast line, differentiated into myotubes, displays robust oscillations in the expression of core clock genes and clock‐controlled genes after synchronization with 50% serum shock (Altıntaş *et al*., 2020).

The molecular clock is calibrated to the diurnal activities of an organism by external entraining signals or zeitgebers. The most well characterized zeitgeber is light, which rapidly phase‐shift clock gene transcription in the SCN and more slowly in peripheral tissues (Kiessling *et al*. 2010). Other zeitgebers that have been hypothesized include food intake and activity. A role for exercise in entraining central and peripheral circadian clocks is becoming increasingly apparent (Gabriel & Zierath, 2019). In rodents, both voluntary access to a running wheel (Maywood *et al*. 1999) and enforced exercise paradigms (Schroeder *et al*. 2012) influence clock gene expression in the SCN. Exercise modulates rhythmic physiological processes such as body temperature and levels of circulating melatonin, which potentially contributes to the diurnal variations in performance (Lewis *et al*. 2018). In the skeletal muscle, exercise induces changes in specific clock genes in rodents (Yamanaka *et al*. 2008; Wolff & Esser, 2012; Ezagouri *et al*. 2019) and humans (Zambon *et al*. 2003; Popov *et al*. 2019). In humans, some of these differences persist in one‐leg exercise paradigms in which one leg was exercised and contralateral leg was used as a resting control (Zambon *et al*. 2003; Popov *et al*. 2019), suggesting that this response may be intrinsic to the exercised muscle.

Exercise is associated with multiple physiological events, including changes in blood flow, sympathetic activity, and plasma hormone and substrate levels (Hawley *et al*. 2014), which are putative modulators of the effect of exercise on central clocks (Tahara *et al*. 2017). However, the mechanism by which exercise modulates expression of skeletal muscle clock genes is unclear. Therefore, the present study aimed to determine whether skeletal muscle contraction could directly influence circadian rhythmicity and uncover the underlying mechanism by which exercise modulates clock gene expression. We investigated changes in clock gene expression in human skeletal muscle in response to acute exercise and compared these responses to *in vitro* contraction models to remove the signals from conflicting zeitgebers such as light and hormonal and sympathetic changes. We exposed C2C12 myotubes to electric pulse stimulation (EPS), which results in visible contractions in myotubes and evokes many of the physiological changes that occur in skeletal muscle during exercise, including effects on energy metabolism, calcium gradients and the expression of exercise responsive genes (Nikolić *et al*. 2016; Pattamaprapanont *et al*. 2016).

Here, we describe that contraction acutely increased *Per2* expression in human skeletal muscle directly following exercise and in mouse soleus muscle and C2C12 myotubes contracted *in vitro*. We suggest that calcium influx from actively contracting muscle cells may be the mechanism by which exercise acutely increases *Per2* expression. Finally, we report that contraction of myotubes *in vitro* acts as a zeitgeber and is able to phase‐shift *Per2* expression. Collectively, our results provide evidence to suggest that contraction, potentially via a calcium‐mediated signalling pathway, directly entrains the intrinsic skeletal muscle clock in response to acute exercise.

## Methods

### Ethical approval

All animal experiments were approved by the Danish Animal Experiments Expectorate (license number 2015‐15‐0201‐00796) and were carried out in accordance with the guidelines of the University of Copenhagen Department of Experimental Medicine, and also conform with the principles and regulations described in Grundy (2015). Use of 2,2,2‐tribromoethanol as an anaesthetic for terminal procedures was approved by the above licence from the Danish Animal Experiments Expectorate.

### Human muscle samples

The present study was carried out in accordance with the recommendations of the Ethic Committee from the Capital Region of Denmark with written informed consent from all subjects. All subjects gave written informed consent in accordance with the *Declaration of Helsinki*, apart from registration in a database. The protocol was approved by the Ethics Committee from the Capital Region of Denmark (reference H‐1‐2013‐064). Sixteen healthy Danish male volunteers, aged between 18 and 27 years old, were recruited to participate in the study. Clinical characteristics have been previously published (16 individuals from cohorts A and B in the untrained state from Ingerslev *et al*. (2018). The participants performed an intense (80% ${\dot{V}}_{O_{2}\max}$) 15 min exercise bout, in the fasted state, in the morning, for which details of have been reported previously (Pattamaprapanont *et al*. 2016). Biopsies were obtained from the vastus lateralis muscle immediately prior to and 60 min following exercise using the Bergström needle technique, and they were immediately snap‐frozen in liquid nitrogen and stored at −80°C for further analysis. RNA was purified from muscle biopsies using an AllPrep kit (Qiagen, Valencia, CA, USA).

### Meta‐analysis of clock gene expression from human exercise studies

Studies of aerobic and resistance exercise in healthy, young, non‐athletes, non‐obese (body mass index <30 kg m^–2^) individuals were selected from the MetaMEx database (http://www.metamex.eu). Acute exercise studies with skeletal muscle biopsy collection occurring within 0–3 h after the exercise bout were selected. Meta‐analysis was calculated for each clock gene with restricted maximum‐likelihood as described previously (Pillon *et al*. 2020).

### *Ex vivo* muscle contraction

Twelve‐week old male C57BL/6NTac (Taconic, Germantown, NY, USA) mice were housed under a 12:12 h light/dark photocycle and maintained on a standard chow diet, with food and water available *ad libitum*. Mice were anaesthetized with an i.p. injection of a 2.5% solution of equal parts 2,2,2‐tribromo ethanol and tertiary amyl alcohol diluted in sterile saline (Sigma, St Louis, MO, USA), at a dose of 16 µL g^–1^ body weight between 15.00 h and 16.00 h (zeitgeber time 9–10) and soleus muscles were isolated from tendon to tendon and tied with silk ties. Muscle isolation was commenced after deep anaesthesia (determined by complete lack of pedal withdrawal reflex) and, directly following muscle isolation, animals were killed by cervical dislocation. Muscles were incubated at 30°C in oxygenated (95% O_2_ and 5% CO_2_) Krebs–Ringer phosphate buffer (117 mm NaCl, 4.7 mm KCl, 2.5 mm CaCl_2_, 1.2 mm KH_2_PO_4_, 1.2 mm MgSO_4_ and 24.6 mm NaHCO_3_) supplemented with 0.1% BSA and 5 mm glucose in a muscle strip myograph system (820MS; Danish Myo Technology, Hinnerup, Denmark). Muscles were tied and placed under tension and allowed to rest for 30 min before they were stimulated to contract using 0.2 ms pulses at 12 V and 20 Hz. Trains of 30 pulses were delivered every 10 s for three rounds of 6 min contraction with 6 min of rest. This protocol induces contractions approximating 30–40% of maximal force production, mimicking high‐intensity aerobic intervals and, in a similar protocol, increased the expression of exercise responsive genes (Barrès *et al*. 2012). Muscle tissue was collected 1 h after the last round of contractions. Muscle was homogenized in 400 µL of RLT buffer (Qiagen) with β‐mercaptoethanol using steel beads and subjected to 2 × 30 s at 30 Hz of disruption using the TissueLyser II (Qiagen) and RNA was extracted using the RNeasy mini kit (Qiagen).

### Cell culture

C2C12 myotubes (#CRL‐1772; American Type Culture Collection, Manassas, VA, USA) were cultured as described previously (Pattamaprapanont *et al*. 2016) and circadian synchronized using 50% horse serum shock for 2 h on day 5 of differentiation. EPS was applied to the cells in culture to induce contractile activity, 12 h after serum shock (12, 18 and 24 h after serum shock in Fig. 3), using the Ion‐optics system C‐dish electrode in a six‐well format. The EPS protocol consisted of 30 min with 1 Hz frequency, 2 ms in duration and 30 V, which induces expression of exercise responsive genes (Pattamaprapanont *et al*. 2016). Differentiated myotubes were treated with pharmacological agents, 12 h after serum shock, to interrogate contraction‐mediated signalling at the following concentrations: forskolin 1 µm (#F3917; Sigma), ionomycin 1 µm (#I3909; Sigma), AICAR 1 mm (#A611700; Toronto Research Chemicals, North York, ON, Canada) or nifedipine 100 µm (#N7634; Sigma), dissolved in DMSO or DMSO (vehicle), added directly to the media, for the appropriate times as indicated. To extract RNA, cells were harvested in RLT buffer with β‐mercaptoethanol and RNA was isolated using the RNeasy mini kit (Qiagen). To extract protein, cells were harvested in RIPA buffer containing protease and phosphatase inhibitors.

### cDNA synthesis and quantitative PCR

cDNA was synthesized using the Bio‐Rad iScript cDNA synthesis kit from 1µg of RNA. A quantitative PCR was performed using conventional Sybr Green chemistry utilizing the primers reported in Table 1. All primers were used at a final concentration of 200 nm. Relative quantification was determined by comparing samples to a standard curve of pooled cDNA or using the ∆∆CT method (in which case efficiency had been previously tested to be 100 ± 10%) for each gene and normalized to the housekeeping genes *18S* (for mouse samples) and *GAPDH* (for human samples). *GAPDH* was chosen for human samples because its expression is stable following endurance exercise (Mahoney *et al*. 2004) and between night and day (Wefers *et al*. 2018) in human skeletal muscle. 18s rRNA was chosen for mouse samples because it remains relatively consistent over the diurnal cycle (Nakao *et al*. 2017) and is routinely used as a housekeeping gene for circadian mouse experiments in skeletal muscle (Kinouchi *et al*. 2018; Altıntaş *et al*. 2020).

**Table 1:** Primer sequences

Gene	Sequence (5'‐ to 3')
Mouse	
*18S*	Forward: AGT CCC TGC CCT TTG TAC ACA
	Reverse: GAT CCG AGG GCC TCA CTA AAC
*Arntl*	Forward: TAG GAT GTG ACC GAG GGA AG
	Reverse: TCA AAC AAG CTC TGG CCA AT
*Per2*	Forward: AAT GGC CAA GAG GAG TCT CA
	Reverse: ATG CTT CCT TCT GTC CTC CA
*Per1*	Forward: TGA AGC AAG ACC GGG AGA G
	Reverse: CAC ACA CGC CGT CAC ATC AA
*Nr1d1*	Forward: GTC TCT CCG TTG GCA TGT CT
	Reverse: CCA AGT TCA TGG CGC TCT
*Cry1*	Forward: AGC GCA GGT GTC GGT TAT GAG C
	Reverse: ATA GAC GCA GCG GAT G GT GTC G
*Per2* promoter (CHIP‐PCR)	Forward: CCA GAA CAA TGT AGC CAC CA Reverse: ACA CTC CCC AAC ACA CAC AA
CHIP negative control region (upstream of *Actb*, chromosome 5)	Forward: CTC TTC CAG CCC TGT TTT TGT G Reverse: ACC TTA AAT CCC AGC ACT CAG G
Human	
*GAPDH*	Forward: GTC AGC CGC ATC TTC TTT TG
	Reverse: TAC GAC CAA ATC CGT TGA CT
*ARNTL*	Forward: TGC CTC GTC GCA ATT GG
	Reverse: ACC CTG ATT TCC CCG TTC A
*PER2*	Forward: GAC ATG AGA CCA ACG AAA ACT GC
	Reverse: AGG CTA AAG GTA TCT GGA CTC TG
*PER1*	Forward: GCC GTG CTG CCT GCT GAT
	Reverse: GGC AGC TGG GTG TGT GCC
*NR1D1*	Forward: CTT CAA TGC CAA CCA TGC AT
	Reverse: CCT GAT TTT CCC AGC GAT GT
*CRY1*	Forward: GCA TCA ACA GGT GGC GAT TT
	Reverse: TGC GTC TCG TTC CTT TCC AA

### Immunoblotting

Cells were harvested in RIPA buffer containing protease (#S8820; SIGMAFAST Protease Inhibitor Cocktail Tablet; Sigma) and phosphatase inhibitors (10 mm NaF, 1 mm Na_3_VO_4_). Protein amounts were normalized after determination of protein concentration using BCA protein assay kit (Pierce, Rockford, IL, USA) in a standard laemmli buffer and subjected to SDS‐PAGE, transferred to polyvinylidene fluoride membranes, blocked in 2% BSA and blotted for phosphorylated cAMP response element‐binding protein (pCREB) (#9198; Ser133) or total CREB (#9197) antibodies (Cell Signaling Technologies, Beverly, MA, USA) at a dilution of 1:1000 and then goat anti‐rabbit IgG‐HRP conjugate (#1706515; Bio‐Rad, Hercules, CA, USA) at a dilution of 1:5000 in 2% BSA. Total CREB was visualized on the same membrane as pCREB after stripping (15 min; Restore Stripping Buffer; Thermo Fisher, Waltham, MA, USA) and re‐probing. Protein loading was determined using Bio‐Rad stain‐free technology.

### Calcium imaging

Differentiated C2C12 myotubes were washed in PBS and incubated in serum‐free, indicator‐free Dulbecco's Modified Eagle's Medium (DMEM) in the presence of 1 µm fluo‐4 acetyloxymethyl ester (#F14201; Thermo Fisher) for 30 min. Before fluorescence measurements were commenced, cells were washed with PBS and incubated in indicator‐free DMEM for 30 min. Live cell imaging was performed on an LSM 780 Confocal microscope (Carl Zeiss, Oberkochen, Germany). Cells were excited with an argon laser and sequential images were taken during the EPS protocol described above.

### Chromatin immunoprecipitation (ChIP)‐PCR

Immediately following the 30 min EPS protocol (and/or treatment with vehicle, ionomycin 1 µm, or nifedipine 100 µm), cells were washed with PBS and fixed in PBS with 1% formaldehyde for 10 min at room temeperature. ChIP was performed as described previously (Williams *et al*. 2020). The antibodies used for ChIP were: pCREB Ser 133 (#9198; Cell Signallng Technologies) and Normal Rabbit IgG (#2729; Cell Signaling Technologies). Quantitative PCR was performed on immunoprecipitated DNA using the primers reported in Table 1.

### Statistical analysis

Data are presented as the mean ± SD or means displaying all data points. Human vastus lateralis and mouse soleus muscle data were tested for normality and analysed by a paired *t* test (normally distributed) or a Wilcoxon matched pairs signed rank test (not normally distributed); *n* indicates the number of individual subjects or animals for these tests. C2C12 experiments were analysed either by the Mann–Whitney test, one or two‐way ANOVA, or corresponding non‐parametric tests, as appropriate; *n* indicates the number of replicates. The rhythmicity analysis presented in Fig. 3 was performed by a two‐way ANOVA with type‐II error assumption as a result of non‐equal group sizes at the same time as considering the main effects ‘group’ as different EPS treatment times and ‘time’ as the measurement time (*expression ∼ group + time*). ANOVA assumptions of univariate normality and homogeneity of variance were not met and the data were power transformed using Tukey's ladder approach. Time points of EPS treatment groups were shifted back to control condition to ensure a proper evaluation of treatment effect. All C2C12 experiments were performed between two to three times on separate days. *P* < 0.05 was considered statistically significant.

## Results

### Contraction acutely increases *Per2* expression in skeletal muscle

**Figure 1:**
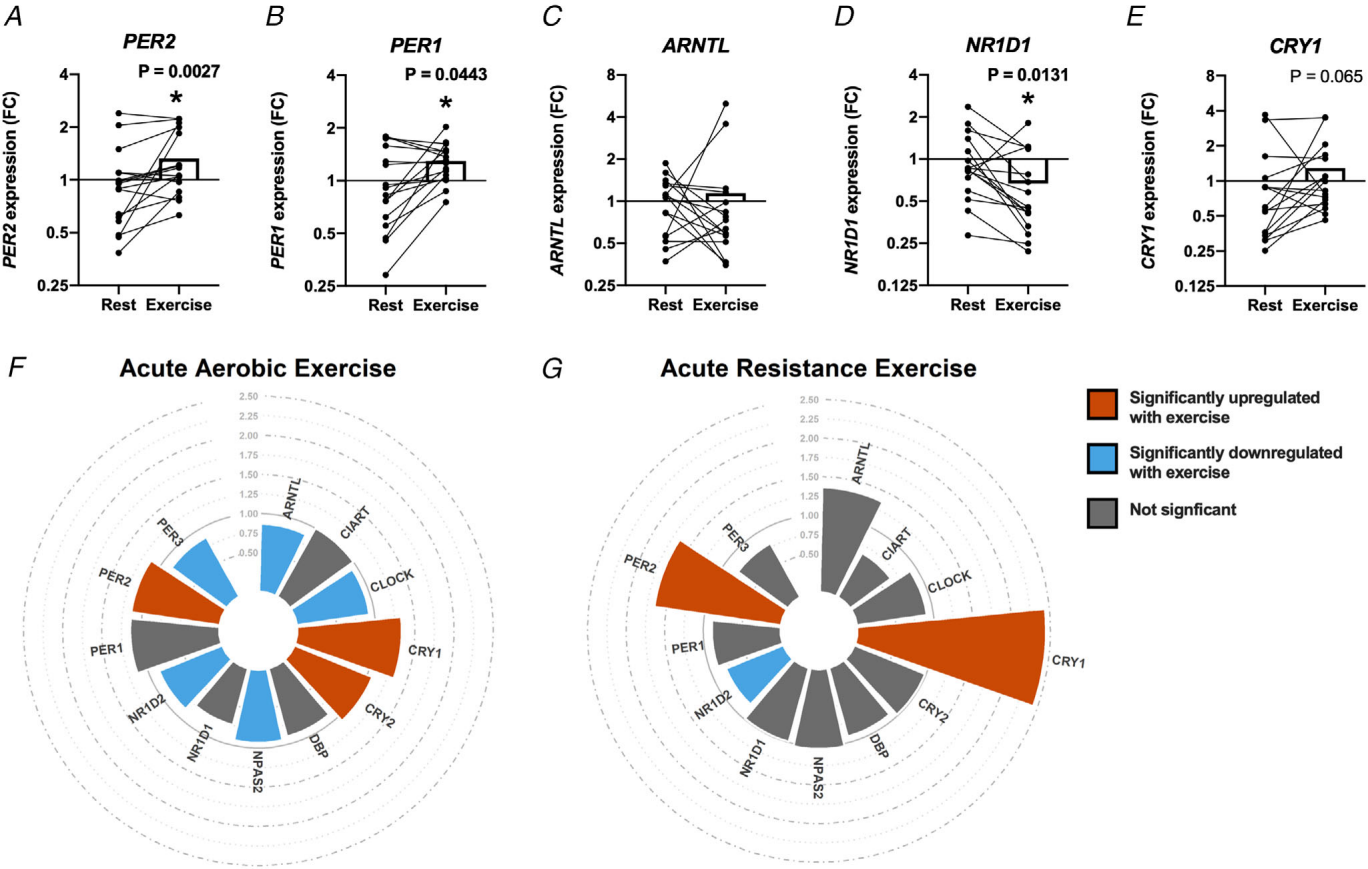
Exercise acutely affects clock gene expression in human skeletal muscle mRNA expression of clock genes from biopsies of human vastus lateralis muscle taken immediately prior to (Rest) and 60 min following 15 min of intense cycling (Exercise) performed on a bicycle ergometer (80% ${\dot{V}}_{O_{2}\max}$). Expression of (*A*) *PER2*, (*B*) *PER1*, (*C*) *ARNTL*, (*D*) *NR1D1* and (*E*) *CRY1* compared to the housekeeping gene *GAPDH* expressed as fold change (FC) compared to the mean of the rested muscle. Analysed by A Wilcoxon matched pairs signed rank test (*n* = 16). ^*^ *P* < 0.05. Open bar represents the mean. Core clock gene expression from human skeletal muscle from publicly available data (http://www.metamex.eu) showing the fold change of the post‐exercise (0–3 h after exercise) timepoint compared to the pre‐exercise timepoint after (*F*) aerobic or (*G*) resistance exercise (*n* = 79 for aerobic exercise and *n* = 50 for resistance exercise).

To determine whether exercise can influence the expression of core clock genes in skeletal muscle, we analysed the expression of several clock genes in the vastus lateralis muscle of young men immediately before and 60 min following an intense 15 min exercise bout (80% ${\dot{V}}_{O_{2}\max}$) on a bicycle ergometer. *PER1* and *PER2* expression was acutely increased by ∼1.5‐fold at 60 min following acute exercise, whereas *NR1D1* expression was decreased (Fig. 1). Conversely, *ARNTL* and *CRY1* expression was not changed following acute exercise (Fig. 1). Publicly available skeletal muscle transcriptomic data from human exercise studies revealed a similar pattern, where acute aerobic and resistance exercise (0–3 h post‐exercise) altered the expression of specific clock genes (Fig. 1 *F* and *G*). Acute exercise induced the expression of *PER2* and *CRY1* in skeletal muscle in both aerobic and (to a greater extent) resistance exercise modalities.

**Figure 2:**
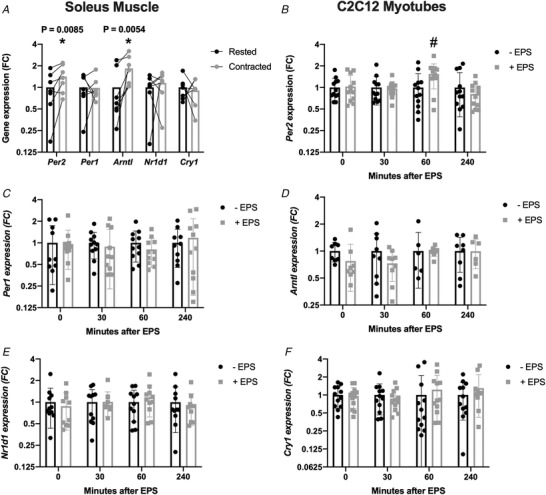
The effect of exercise on *Per2* expression can be recapitulated in skeletal muscle by *in vitro* contraction *A*, mRNA expression of *Per2*, *Per1*, *Arntl*, *Nr1d1* and *Cry1* from contracted mouse soleus muscles compared to the housekeeping gene *18S rRNA*. One soleus muscle from each mouse was contracted for 30 min and then further incubated for 60 min and then harvested with the rested contralateral muscle. Pairwise comparison from the rested *vs*. contracted muscle, with relative expression depicting the fold change (FC) compared to the mean of the rested muscle, analysed by individual paired *t* tests (*n* = 7). mRNA expression of (*B*) *Per2*, (*C*) *Per1*, (*D*) *Arntl*, (*E*) *Nr1d1* and (*F*) *Cry1* from EPS‐treated C2C12 myotubes compared to the housekeeping gene *18S*, 12 h after serum‐shock after a 30 min contraction protocol, with relative expression compared to non‐contracted control cells at each timepoint, analysed by individual Mann–Whitney tests corrected for multiple comparisons (*n* = 6–12). ^*^ *P* < 0.05. ^#^ *P* = 0.0242. AdjP = not significant. Data are presented as the mean ± SD.

To account for the possibility of changes in clock expression as a result of sampling time and to isolate the contribution of muscle contraction *per se* to this effect, two separate *in vitro* models of muscle cell contraction were used. In the first model, we performed *ex vivo* contractions of isolated mouse soleus muscle, comparing the expression of circadian clock genes between the rested and contracted muscle from the same animal 60 min after a 30 min contraction stimulus. *Per2* expression was increased by ∼1.4 fold 60 min after the *ex vivo* muscle contraction procedure (Fig. 2 *A*). Additionally, *ex vivo* contraction increased *Arntl* expression, whereas *Per1*, *Nr1d1* and *Cry1* were unaffected (Fig. 2 *A*). We then performed a time course study to determine the effect of EPS‐mediated contraction on gene expression. C2C12 myotubes were harvested 0, 30, 60 and 240 min following a 30 min EPS stimulus. *Per2* expression (Fig. 2 *B*), but not the expression of *Per1*, *Bmal1*, *Nr1d1* or *Cry1* (Fig. 2 *C–F*), was increased by ∼1.5‐fold, 60 min following EPS; however, *Per2* expression returned to the unstimulated level 4 h post contraction (Fig. 2 *B*; although, after adjusting for multiple comparison testing, this did not reach significance).

### Contraction induces a phase‐shift in the rhythmicity of *Per2* expression in muscle cells *in vitro*

**Figure 3:**
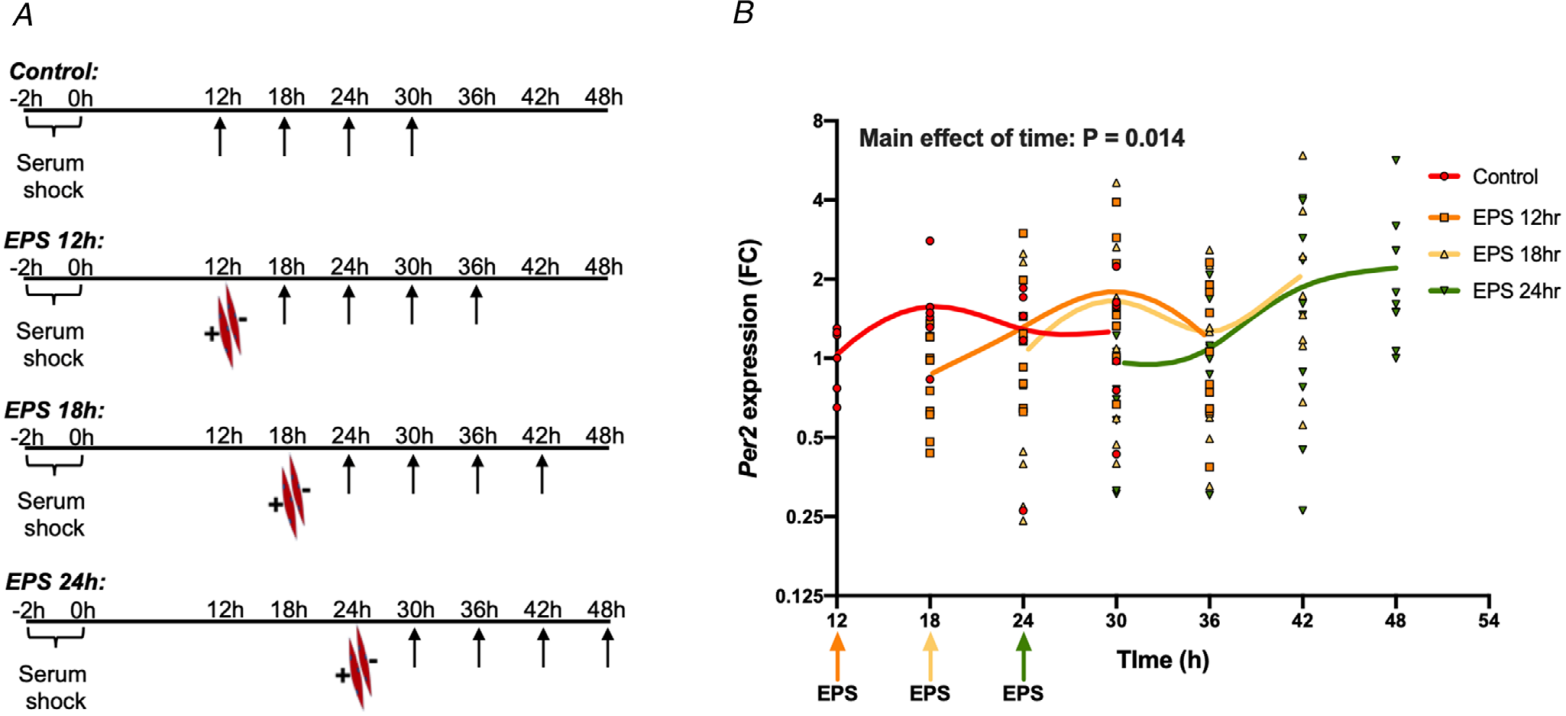
Contraction in C2C12 myotubes phase‐shifts *Per2* rhythmicity *A*, schematic of the experimental protocol in which C2C12 myotubes were synchronized by serum‐shock and then EPS was performed at either 12, 18 or 24 h after circadian synchronization and cells were harvested for RNA every 6 h after EPS or control conditions. *B*, myotubes were subjected to contraction as described in (*A*) and *Per2* expression was compared with the housekeeping gene *18S rRNA*. Relative expression depicting fold change (FC) compared to the mean of control condition at 12 h after circadian synchronization (*n* = 5–10). Data were power transformed using Tukey's ladder of power approach and analysed by two‐way ANOVA (without interactions) using type‐II error assumption. Time points of EPS treatment groups were shifted back to control condition to make a proper evaluation of treatment effect. Data are presented as a spline going through the mean at each timepoint within each group.

Because *Per2* expression was induced after exercise in human skeletal muscle as well as after contraction in both *in vitro* contraction models used, we further investigated the mechanisms by which *Per2* expression was influenced by skeletal muscle contraction. To determine whether the acute contraction‐mediated increase in *Per2* expression is associated with longer term differences in circadian rhythmicity, we subjected synchronized C2C12 myotubes to a single 30 min bout of EPS 12, 18 or 24 h after circadian synchronization (serum shock). Cells were harvested every 6 h for 24 h after each bout of EPS (Fig. 3 *A*). When cells were exposed to EPS at any time point, this induced a phase‐shift in *Per2* oscillation to its low expression starting point (Fig. 3 *B*).

### Modulating intracellular calcium levels alter *Per2* expression

**Figure 4:**
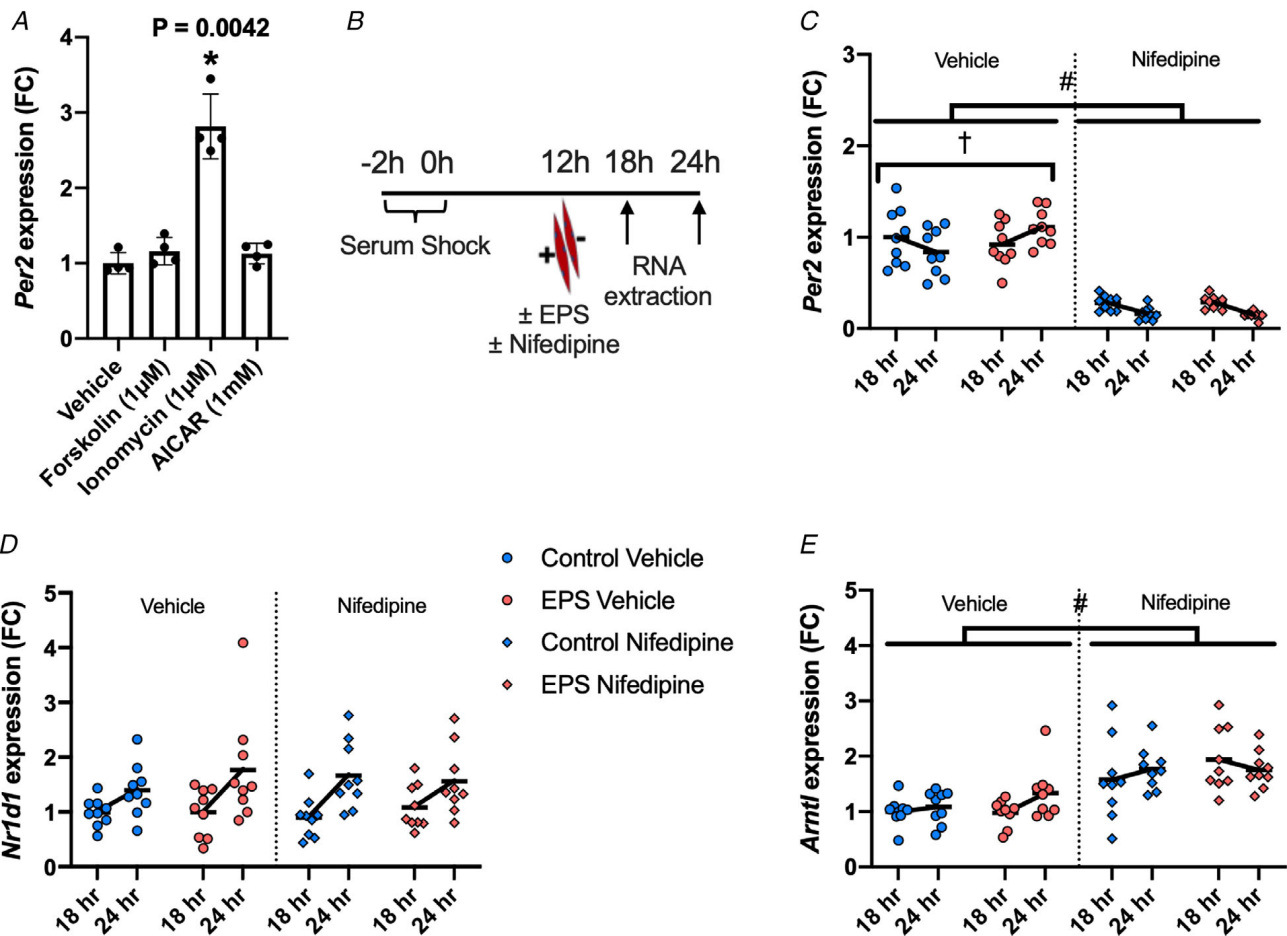
Modulating cellular calcium levels affects the expression of *Per2* and its induction by contraction *A*, mRNA expression of *Per2* in C2C12 myotubes (12 h after serum‐shock) after treatment with vehicle (DMSO), 1 µm forskolin, 1 µm ionomycin or 1 mm AICAR for 90 min compared to the housekeeping gene *18S rRNA*, with relative expression compared to the mean of the vehicle condition. Analysed by a Kruskal–Wallis test with Dunn's *post hoc* test comparing all treatments with vehicle (*n* = 4). *B*, simplified schematic of the experimental design for results presented in (*C*) to (*E*). mRNA expression of (*C*) *Per2*, (*D*) *Nr1d1* and (*E*) *Arntl* in synchronized C2C12 myotubes treated with or without EPS and 100 µm nifedipine or vehicle (DMSO) at 12 h after serum‐shock harvested at 18 and 24 h after serum‐shock, with relative expression compared to the mean of the control‐vehicle condition. Analysed by two‐way ANOVA for an interaction between EPS and time separately for both the vehicle and nifedipine conditions (*n* = 9). Effect of nifedipine was analysed by the Mann–Whitney test of all vehicle treated cells compared to all nifedipine treated cells (*n* = 36). ^*^ *P* < 0.05 compared to vehicle control. ^†^ *P* = 0.0465 EPS/time interaction. ^#^ *P* < 0.0001 effect of nifedipine. Data are presented as the mean ± SD.

To explore mechanisms by which contraction increases *Per2* expression, we treated C2C12 myotubes with several agents that mimic the effects of exercise on signal transduction using concentrations of the compounds that are commonly used with *in vitro* skeletal muscle cell models (Carter & Solomon, 2018). We tested the effect of forskolin (1 µm), which raises cAMP levels, the Ca^2+^ ionophore, ionomycin (1 µm) and the AMPK agonist AICAR (1 mm) on *Per2* expression in C2C12 myotubes after 90 min of treatment. Interestingly, treatment with ionomycin, but not forskolin or AICAR, increased *Per2* expression by ∼2.5 fold (Fig. 4 *A*). To confirm that EPS induced changes in intracellular calcium levels in C2C12 myotubes, we performed EPS on C2C12 myotubes pre‐treated with the calcium indicator, fluo‐4, which fluoresces in the presence of calcium, and we visualized the contracting cells using fluorescence microscopy. Cells that were exposed to EPS displayed visible flashes of fluorescence coincident with the electrical pulses, indicating increased cytoplasmic calcium levels in response to contraction (see Supporting information, Fig. S1).

We next performed an abbreviated experiment in which we treated circadian synchronized C2C12 myotubes in the absence or presence of nifedipine (calcium channel blocker) or EPS, 12 h after serum shock, and then monitored *Per2* expression at 18 and 24 h after serum shock (Fig. 4 *B*). We used a dosage of nifedipine (100 µm) that has been previously used in C2C12 myotubes to reduce contraction‐mediated calcium flux (Porter *et al*. 2002). Nifedipine had no visible effects on cell viability in differentiated cells; however, we did note that EPS‐mediated contraction was compromised in these cells, as indicated by fewer visible contractions, confirming the efficacy of nifedipine with respect to reducing calcium flux (data not shown). We assessed the oscillatory behaviour of *Per2* by calculating the slope of gene expression from 18 to 24 h. We chose these timepoints because C2C12 myotubes display different slopes of *Per2* expression between 18 to 24 h when EPS is performed at 12 h post serum shock (Fig. 3 *B*). EPS caused a significant change in the slope of gene expression in vehicle treated cells compared to the control cells without stimulation (Fig. 4 *C*). Nifedipine treatment reduced *Per2* expression and abolished the effect of EPS on *Per2* expression. Conversely, *Nr1d1* showed a difference in expression with time, but no difference with nifedipine treatment or EPS (Fig. 4 *D*), whereas *Arntl* expression was not altered by either time or EPS, although it was increased by nifedipine treatment (Fig. 4 *E*).

### Contraction induces CREB phosphorylation and binding to the *Per2* promoter

**Figure 5:**
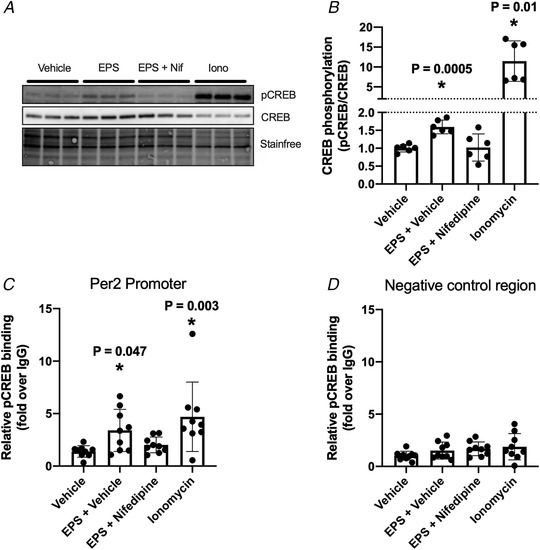
Contraction and increasing cytosolic calcium increase phosphorylation of CREB and its binding to the *Per2* promoter C2C12 myotubes (12 h after serum‐shock) were harvested immediately after a 30 min EPS contraction protocol and incubated in the absence or presence of nifedipine (100 µm) or after a 30 min incubation with vehicle (DMSO) or ionomycin (1 µm) and were harvested separately for immunoblotting or ChIP‐PCR. *A*, representative immunoblot. *B*, quantification of phosphorylated (Ser133) and total CREB protein, analysed by one‐way ANOVA with Dunnett's *post hoc* test comparing all conditions with vehicle (*n* = 6). ChIP‐PCR of the (*C*) *Per2* promoter region and (*D*) a negative control region upstream of the ß‐actin promoter utilizing the pCREB Ser133 antibody and normal rabbit IGG as an antibody control, analysed by a Kruskal–Wallis test with Dunn's *post hoc* test comparing all conditions with vehicle (*n* = 9). ^*^ *P* < 0.05. Data are presented as the mean ± SD.

To determine the mechanism by which calcium levels influence *Per2* expression, we investigated the phosphorylation status of CREB. pCREB binds to cAMP response elements (CREs) in promoters of period gene isoforms in fibroblasts (Pulivarthy *et al*. 2007), PC12 neuroblasts (Impey *et al*. 2004) and the SCN (Tischkau *et al*. 2003). In C2C12 myotubes, EPS acutely increased phosphorylation of CREB, whereas nifedipine treatment prevented this increase (Fig. 5 *A* and *B*). Ionomycin treatment produced an expected robust increase in CREB phosphorylation. Mirroring these results, EPS and, to a greater extent, ionomycin treatment increased pCREB antibody‐mediated pull‐down of the *Per2* promoter region (Fig. 5 *C*). This was not the case with a negative control region (upstream of the β‐Actin promoter) (Fig. 5 *D*). Taken together, these data show that contraction‐mediated increases in cytosolic calcium leads to increases in phosphorylated CREB, which binds to CREs on the *Per2* promoter. Our results suggest a role of calcium‐mediated CREB binding in the positive regulation of *Per2* expression.

## Discussion

Evidence obtained in studies conducted in mice (Ezagouri *et al*. 2019; Sato *et al*. 2019) and humans (Savikj *et al*. 2019) suggests that exercise performed at different times of day results in diverse metabolic outcomes. However, there is still no consensus on the mechanisms that underlay these differences. Because metabolism is strongly influenced by the intrinsic core clock machinery (Dyar *et al*. 2014), one possibility is that exercise‐induced changes in the skeletal muscle core clock machinery may alter the expression of clock‐controlled genes and impact metabolic processes. Although several reports suggest exercise‐induced alterations in specific skeletal muscle clock genes (Zambon *et al*. 2003; Yamanaka *et al*. 2008; Wolff & Esser, 2012), determining the mechanisms driving these changes has been a challenge because exercise influences many physiological processes. We hypothesized that skeletal muscle contraction during exercise was partly responsible for exercise‐mediated changes in clock gene expression. To address this question, we utilized an *in vitro* contraction model to isolate the contribution of contraction from other elements associated with exercise in the working muscle.

We found that *PER1* and *PER2* were increased and *NR1D1* was decreased in human skeletal muscle 1‐h post‐exercise. Similarly, a meta‐analysis of publicly available data revealed that *PER2* and *CRY1* expression in human skeletal muscle was acutely increased after both aerobic and resistance exercise, with resistance exercise having a larger effect. Interestingly, only *Per2* was similarly increased in contracting mouse skeletal muscle and cultured C2C12 myotubes *in vitro*. Treadmill running acutely increases *Per1* and *Per2* expression in mouse plantaris muscle 1 h following the exercise bout (Saracino *et al*. 2019). This increase in *Per1*, but not *Per2* expression, was attributed to exercise‐secreted hormones such as epinephrine. Because *PER2* expression is acutely increased in skeletal muscle after one‐legged exercise regimes (Zambon *et al*. 2003; Popov *et al*. 2019) and by contraction *in vitro*, systemic hormonal cues probably do not play a role, suggesting that expression of *Per1* and *Per2* isoforms are driven by separate exercise stimuli. Similarly, acute resistance exercise, but not *in vitro* contraction, led to a substantial up‐regulation of *CRY1* expression in skeletal muscle, suggesting that *CRY1* expression is regulated by a separate exercise stimulus, and not muscle contraction *per se*.

In synchronized C2C12 myotubes that are removed from conflicting zeitgebers, we show that the induction of *Per2* by contraction is transient because it disappears after 4 h. However, this transient increase in *Per2* expression at 1 h post‐EPS is followed by a decrease in *Per2* expression to its lowest point 6 h after EPS, resetting its cycle. Interestingly, contraction phase‐shifted *Per2* rhythmicity in C2C12 myotubes at multiple timepoints of the circadian cycle. A similar ability of exercise to modulate *Per2* rhythmicity has been reported in mouse skeletal muscle explants taken from mice with access to voluntary running wheels or forced treadmill running, which display shifted *Per2* rhythms for multiple days *ex vivo* (Wolff & Esser, 2012).

Contraction of C2C12 myotubes cells alters the rhythmicity of *Arntl*, as evidenced by a small (∼1 h) contraction‐induced phase‐shift (as determined by monitoring of *Arntl* ‐luciferase luminescence) (Kemler *et al*. 2020). Thus, either *Arntl* and *Per2* are regulated differently by contraction or, alternatively, the resetting effect of contraction on *Per2* rhythmicity as described in our study may only be at the transcript level and the translated protein may have a different rhythmicity. By contrast to our results, *Per2* expression was reduced immediately after 1 h of contraction in C2C12 myotubes performed at 22 and 28 h after synchronization (Kemler *et al*. 2020). Potentially, this discrepancy is a result of the time after contraction (1 h in the present study; immediately after contraction in Kemler *et al*. 2020), time after synchronization (12 h in the present study; 22 or 28 h after synchronization in Kemler *et al*. 2020) or method of synchronization (serum shock in the present study; dexamethasone in Kemler *et al*. 2020). However, overall, the report by Kemler *et al*. (2020) supports the hypothesis that skeletal muscle contraction can entrain core clock genes.

The effect of EPS to phase‐shift clock gene expression in cultured cells is not entirely surprising because the rhythmicity of cultured cells is synchronized by multiple signals, including serum shock, dexamethasone, epidermal growth factor and ionomycin (Izumo *et al*. 2006). However, in an *in vivo* setting, exercise probably acts as a relatively weak zeitgeber compared to light, and therefore the ability of acute exercise or contraction to influence clock rhythmicity over a long period is probably limited. Supporting this, a recent study performed by our group found few differences in the rhythmicity of core clock genes in mouse skeletal muscle for 24 h following acute treadmill exercise at two different times of day compared to skeletal muscle from non‐exercised mice investigated at the same times (Sato *et al*. 2019). However, immediately following exercise, the expression of several clock genes was transiently increased, and this was dependent on the time of day when the exercise bout was performed. Correspondingly, one limitation of the present study is that the acute transcriptional response to contraction was tested at only one timepoint in human and mouse skeletal muscle. Potentially, contraction‐mediated changes in clock gene expression may rely on a certain expression level, which may occur only during a distinct period for each respective gene over the circadian rhythm.

Calcium flux has a well‐established role in the entrainment of circadian rhythms in the neurons of the SCN. In particular, *Per2* expression is dependent on functional calcium transport in SCN neurons (Lundkvist *et al*. 2005) and fibroblasts (Noguchi *et al*. 2012). We provide novel evidence indicating that alterations in cellular calcium flux mediated by contraction may be partly responsible for the entrainment of specific core clock genes in skeletal muscle. Increasing cytosolic calcium with the calcium ionophore ionomycin increased *Per2* expression, whereas blocking calcium transport with nifedipine decreased *Per2* expression, increased *Arntl* expression (potentially as a result of the large decrease in *Per2* expression) and abolished the effect of EPS on *Per2* expression. However, because nifedipine treatment reduced the appearance of visible contractions in EPS‐stimulated cells, this may be the result of an effect of nifedipine on contraction *per se* rather than on calcium flux itself. Because calcium flux is necessary for muscle contraction, it is difficult to isolate the contribution of one from the other.

CREB phosphorylation plays a role in regulating the response of the SCN clock to stimuli such as light (Gau *et al*. 2002; Wheaton *et al*. 2018). A CRE has been identified in the *Per2* promoter (Impey *et al*. 2004) and *Per* isoforms are driven by CREB binding to CREs in promoter regions (Tischkau *et al*. 2003; Pulivarthy *et al*. 2007). We provide evidence indicating that, in C2C12 myotubes, CREB phosphorylation at Ser 133 is responsive to increasing cytosolic calcium through EPS stimulation and ionomycin treatment. Furthermore, our ChIP‐PCR experiments suggest that phosphorylated CREB binds to the *Per2* promoter, and this effect is enhanced after either EPS or ionomycin treatment. Although we find that, in C2C12 cells, CREB is phosphorylated immediately after contraction, in human skeletal muscle, CREB phosphorylation decreases immediately following exercise (compared to the rested leg) (Widegren *et al*. 1998), with an increase not occurring until 3 h post‐exercise (Egan *et al*. 2010; Stocks *et al*. 2019). Therefore, the exact time‐course for CREB phosphorylation after exercise is unclear, indicating that other factors occurring in the human setting, such as exercise intensity and duration, as well as differences in secreted factors, may be involved in regulating the expression of *Per2* after exercise. Although we focused on the Ser 133 phosphorylation site of CREB, because of the availability of a ChIP‐verified antibody, it is also possible that other CREB phosphorylation sites may play a role in this process, such as Ser 142, which is involved in the regulation of light‐induced phase‐shifts in the SCN (Gau *et al*. 2002).

The physiological role of exercise on clock components in skeletal muscle is unclear. The acute exercise‐induced changes in skeletal muscle clock genes probably do not affect long‐term circadian rhythmicity in mammals that have a normal sleep/wake cycle aligned with the light. Potentially, longer scheduled exercise routines have a greater effect on the clock and it is possible that, in situations in which there is a divergence between the central and peripheral clock, such as with jet‐lag, exercise may have a fine‐tuning effect to rapidly align the peripheral clock with the organism's behaviour. Along these lines, aerobic exercise at different times of the day had some capacity to phase‐shift diurnal melatonin secretion (Youngstedt *et al*. 2019). Potentially, when adapting to a new light:dark schedule, the ability of contraction to entrain the muscle clock may allow the tissue to more quickly adapt to behavioural changes.

In conclusion, the results obtained in the present study add to an increasing body of evidence indicating that exercise alters the transcription of skeletal muscle clock genes. Evidence is also provided indicating that a proportion of this effect is directly a result of muscle contraction *per se*. Additionally, we show that the ability of contraction to alter *Per2* expression is dependent on contraction‐mediated changes in cytosolic calcium content. This work advances our understanding of how non‐light zeitgebers influence circadian rhythmicity in peripheral tissue, with applications towards delineating the links between lifestyle factors, circadian rhythms and metabolic disease.

## Additional information

### Competing interests

The authors declare that they have no competing interests.

### Author contributions

LS, RCL and RB were responsible for the study conception and design. LS, AA, RCL, AE, PP, JV, NJP and RB were responsible for data acquisition, as well as analysis and interpretation. LS, RCL, JRZ and RB were responsible for drafting and revising the article. All authors approved final version of the manuscript submitted for publication and agree to be accountable for all aspects of the work.

### Funding

The Novo Nordisk Foundation Center for Basic Metabolic Research is an independent research Center at the University of Copenhagen, partially funded by an unrestricted donation from the Novo Nordisk Foundation (NNF18CC0034900). This work was funded by a Novo Nordisk Foundation Challenge Grant (NNF14OC0011493) and partly supported by a research grant from the Danish Diabetes Academy, which is funded by the Novo Nordisk Foundation (NNF17SA0031406).

## Supporting information


**Video S1**. Live cell imaging video of individual C2C12 myotubes treated with the cytosolic calcium fluorophore, fluo‐4, undergoing EPS‐mediated contraction.

**Statistical Summary Document**

## Data Availability

The data that support the findings of this study are available from the corresponding author upon reasonable request.
